# Non-pharmaceutical strategies win coronavirus disease 2019 battle in New Zealand

**DOI:** 10.4102/jamba.v12i1.1010

**Published:** 2020-12-11

**Authors:** Godwell Nhamo, Gwendoline Q. Kandawasvika, Mncengeli Sibanda

**Affiliations:** 1 Institute for Corporate Citizenship, College of Economic and Management Sciences, University of South Africa, Pretoria, South Africa; 2 Department of Paediatrics and Child Health, College of Health Sciences, University of Zimbabwe, Harare, Zimbabwe; 3 Department of Public Health Pharmacy and Management, School of Pharmacy, Sefako Makgatho Health Sciences University, Pretoria, South Africa

**Keywords:** COVID-19, flatten the curve, influenza pandemic plan, lockdown, New Zealand

## Abstract

This literature-based article found that on 08 June 2020, New Zealand claimed victory over coronavirus disease 2019 (COVID-19) mainly because of effective non-pharmaceutical strategies and interventions that included a hard lockdown. The response was informed by the country’s Influenza Pandemic Plan (although without criticism), which was updated in 2017, and the swift responses from political leadership and other key stakeholders. Strategies instituted included the proclamation of urgent precautionary measures leading to border closures, issuing of a 3-month-long COVID-19 notice under the *Epidemic Preparedness Act* 2006, the proclamation of the COVID-19 Elimination Strategy and the Initial COVID-19 Māori Response Action Plan, which incorporated COVID-19 Alert Levels that facilitated stepwise easing of the hard lockdown. The non-pharmaceutical strategies seem to have worked again, even as the second wave of COVID-19 infections returned in August 2020 through an Auckland cluster. Hence, the New Zealand case remains one that the world can draw lessons from, although not perfect.

## Introduction

The gradual easing of lockdowns in most countries including New Zealand kicked in from May 2020. New Zealand’s non-pharmaceutical strategies towards coronavirus disease 2019 (COVID-19) resulted in the country declaring itself COVID-19 free on 08 June 2020 (Boston Globe 2020) – a global first. Non-pharmaceutical strategies include measures not involving vaccines and medical treatments. Although most of the strategies employed draw from past epidemics and pandemics such as the 1918 influenza – Spanish flu (Nickol and Kindrachuk 2019), A(H1N1) 2009, the severe acute respiratory syndrome (SARS), the Middle East respiratory syndrome (MERS) and Ebola (Elhakim et al. 2019), it is the swift manner in which New Zealand acted that deserves space in this article and for the world to draw lessons from. However, it should be noted that the country did not adopt mass-masking or mandate masking until August 2020 after the outbreak of the second wave (The Guardian 2020).

**FIGURE 1:**
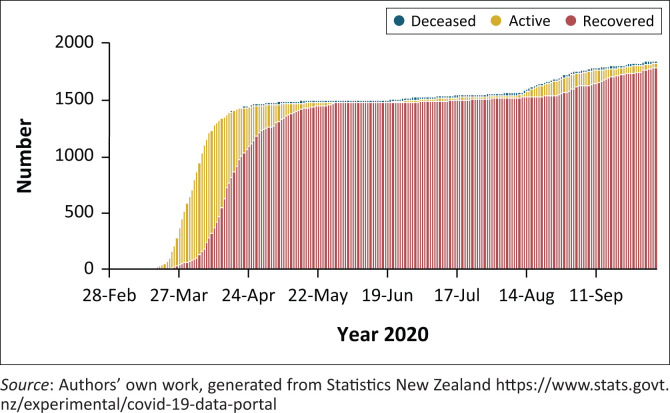
Coronavirus disease 2019 cases status in New Zealand as of 05 October 2020.

As this article was being finalised on 05 October 2020, COVID-19 global infections were about 35.24 million, with deaths just over 1.038 million as per data from the John Hopkins University (2020). There were also worries that Africa is likely to become the next epicentre, making the use of non-pharmaceutical strategies even more critical (Loembé et al. 2020). Lazarus et al. (2020) call this ‘the tried and tested public health measures’ commonly used in controlling disease and pandemic outbreaks. New Zealand had 1499 confirmed cases, 25 deaths and 1855 confirmed and probable cases as of 05 October 2020 (Ministry of Health 2020a). A total of 40 cases were still active. The country reported Patient Zero on 28 February 2020, with the infection curve rising sharply to about 1106 cases by 06 April 2020, before the curve started to ‘flatten’ and maintaining infection figures of about 1500 from 22 May 2020 to 14 June 2020. The trends and daily distribution of COVID-19 cases in New Zealand are presented in Figure 1.

There is also interesting epidemic data from COVID-19 cases in New Zealand. Of the reported cases as of 05 October 2020 (1855 in total), 55% were women and 45% were men (Ministry of Health 2020a). In addition, the bulk of the reported cases (39%) were imported. This was followed by locally acquired cases (36%) and those of imported related nature stood at 25%. Concerning deaths, there were no such cases in the 0–49 years age groups. However, of the 25 deaths reported, two fell in the 50–59 years age cohort, three were in the 60–69 years age cohort, seven in the 70–79 years age group, eight in the 80–89 years age group and five in the 90+ years age cohort.

Given the above discussion, this article sets an objective to determine the key interventions that made New Zealand one of the unique countries to successfully overcome the COVID-19 pandemic, especially from both the first and second waves of infections. The article also seeks to establish lessons that other countries can draw from the New Zealanders’ pandemic fight, with the view to enhance ongoing non-pharmaceutical interventions, especially for countries likely to receive the vaccines and treatments late once found and approved.

## Non-pharmaceutical strategies in epidemic and pandemic fight

Without current known vaccines and treatments, the use of non-pharmaceutical strategies in the fight against the severe acute respiratory syndrome coronavirus 2 (SARS-CoV-2), which causes the COVID-19 disease, remains the most viable option. The strategies further support provisions of the United Nations Sendai Framework on Disaster Risk Reduction (DRR) commissioned in 2015 and lasting till 2030 (United Nations Office for Disaster Risk Reduction 2015). From the Sendai Framework, aspects of the disaster management cycle emerge, including preparedness and early warning, relief and response, as well as recovery and reformation. Furthermore, the Sendai Framework embeds the concept of building back better after such global pandemics as COVID-19 and other disasters.

Using non-pharmaceutical measures to fight pandemics is not new. During the 1918 influenza, the Mexican government set up sanitary brigades (Alexander 2019) that went to each state establishing non-pharmaceutical behavioural protocols. These included health education and awareness campaigns, enforcing prescriptive regulations on public gatherings and personal mobility on trains. In the modern-day language of ‘flattening’ pandemic curves (Kenyon 2020), the government further irrigated streets with water, inspected ships and trains and also advised on isolation, quarantine and instituted curfews. Those that broke the regulations were penalised as appropriate. Similar strategies, including lockdowns, were instituted in the United States of America (Keeling 2020).

Contact tracing and mass testing were some of the non-pharmaceutical strategies that gained popularity during MERS outbreaks in South Korea in 2015 and were repeated for COVID-19 (Moatti 2020). South Korea also reported a measure of success from non-pharmaceutical responses that included reforming epidemic preparedness systems and health regulatory frameworks after MERS, expanding rapid tests and information sharing. Other measures involved setting up specialised clinics and the modification of personal protective equipment by hospitals, with healthcare staff reporting related COVID-19 symptoms (Kang et al. 2020). Mass testing requires a network of accredited laboratories and related infrastructure, including readily available testing kits, and many countries did not have such resources. However, contact tracing through mobile apps has raised ethical issues concerning the intrusion into privacy as their utilisation grew in Australia, China, Israel, Singapore, South Korea and other countries (Sharma & Bashir 2020).

Focusing on the United Kingdom, Davies et al. (2020) found that lockdown and stay-at-home periods brought *R*_0_ near or below 1. Stay-at-home orders also identified older age groups and people living with underlining conditions such as diabetes, high blood pressure and associated respiratory problems. Davies et al. (2020) established that extreme non-pharmaceutical strategies would be needed to bring COVID-19 under control and avoid surges in deaths and ramped up demand for hospital beds, particularly those in intensive care units (ICUs). Drawing from Wuhan data, Colbourno (2020) reported that appropriate physical distancing had the potential to delay COVID-19 peak by up to months. In addition, health promotion, information and communication about COVID-19 were important as the first shield where populations were asked to regularly wash their hands, as well as practise cough and sneezing etiquette. Health promotion remains a formidable strategy for both communicable and non-communicable diseases and individuals should also take responsibility. Apart from the measures highlighted already, work from Spain reveals that effective COVID-19 governance and coordination between national and state or regional levels is crucial as this permits the rapid deployment of resources where they will be needed most (Paakkari & Okan 2020). Other building blocks identified in the non-pharmaceutical strategies in Spain include financing, service delivery, availability of medical equipment and effective information sharing and communicating one streamlined government agenda.

Whilst the administrative situation and enforcement of social distancing regulations and COVID-19 strategies may work effectively in developed countries, this may not be the case in developing countries such as Zimbabwe. In a recent study by Musarandega and Chitongo (2020), it was found that rural livelihoods are by their very nature socially intertwined, prohibiting social distancing. This is so in direct opposite to the fact that most households in rural Zimbabwe are physically distanced. Practices such as the chief’s granary scheme have it that community members gather to work in the chief’s fields to produce strategic communal food that will be used during times of disasters, such as droughts or floods. In addition, communities graze their livestock together and fetch water from common sources such as boreholes, springs and communal taps. There are also many more communally owned properties and resources that act as barriers to the need of social distancing measures. Also, measures such as curfews are ineffective as governments do not have adequate policing resources for these sparsely populated areas. For effective non-pharmaceutical strategies to work in rural communities such as Biriiri in Chimanimani, the authors recommend ‘the adoption of a cultural framework that utilises traditional governance to facilitate social distancing without compromising the livelihoods of people in remote rural settings’ (Musarandega & Chitongo 2020:309).

The next section focuses on detailing what New Zealand did well in order to succeed in fighting the first and second COVID-19 infections waves.

## What did New Zealand do right to win the battle?

The choice of words to use the COVID-19 battle as opposed to war is deliberate, as the war is still on. New Zealand instituted four major COVID-19 non-pharmaceutical strategies informed by the updated Influenza Pandemic Plan (IPP) and proactive political engagement and leadership: (1) the proclamation of urgent precautionary measures on 14 March 2020 leading to border closures, (2) issuing of a 3-month-long COVID-19 notice under the *Epidemic Preparedness Act* 2006 (23 March 2020), (3) the proclamation of COVID-19 Elimination Strategy (23 March 2020) and (4) the Initial COVID-19 Māori Response Action Plan of 16 April 2020 that incorporated the COVID-19 Alert Levels (Table 1) that facilitated controlled and stepwise opening of the lockdown.

**TABLE 1:** Summary of New Zealand’s coronavirus disease 2019 alert levels.

Alert level	Prevalence of COVID-19 infections	Risk assessment
Level 4: Lockdown	Likely that disease is not contained	Sustained and intensive community transmission is occurring Widespread outbreaks
Level 3: Restrict	High risk – the disease is not contained	Multiple cases of community transmission occurring Multiple active clusters in multiple regions
Level 2: Reduce	The disease is contained, but the risk of community transmission remains	Limited community transmission could be occurring Active clusters in more than one region
Level 1: Prepare	The disease is contained in New Zealand	COVID-19 is uncontrolled overseas Sporadic imported cases Isolated local transmission could be occurring in New Zealand

*Source*: Adapted from New Zealand Government, 2020a, *New Zealand COVID-19 alert levels summary*, viewed 05 October 2020, from https://covid19.govt.nz/assets/resources/tables/COVID-19-alert-levels-summary.pdf.

In 2017, New Zealand updated and modernised its 2010 IPP (Legido-Quigley et al. 2020). The IPP is a framework for action spelling out all-of-government interventions that need to be taken in preparation for and responding to any influenza pandemic. The IPP makes it clear that pandemics are unpredictable in terms of their timing, severity and population groups most impacted. Hence, the revised IPP was set up in a manner that permits its rapid adoption and application to any pandemic regardless of the nature of the virus and its severity. To this end, one can conclude that part of the success of COVID-19 pacification by New Zealand is attributed to this pandemic readiness framework. The IPP incorporated lessons from the pandemic influenza A(H1N1) 2009 response. Summing up the key features of the IPP, the New Zealand Director General of Health indicated that it (New Zealand Government 2017:iii):

[*R*]eflects the sophistication of a third generation, risk-based plan that promotes collaboration across all levels of government, agencies and organisations when planning for, responding to and recovery from a pandemic event.

**FIGURE 2:**
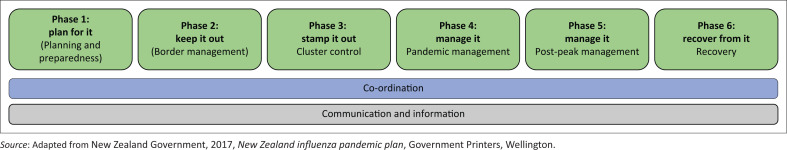
Pandemic response phases and interventions as per the Influenza Pandemic Plan.

The IPP presents six phases (Figure 2). The lesson coming up here for the other governments is to prepare for pandemics ahead of time.

However, the IPP is not a perfect document as there are also criticisms levelled against it. Strang (2020) reported some epidemiologists working in the public health space at the University of Otago and King’s College London suggesting that the country needs a new generic pandemic plan. Such a proposed new plan should cater for worst-case scenarios. To this end, the epidemiologists suggested that the updated 2017 IPP was not fit for purpose. Three key shortfalls were, therefore, singled out: (1) the assumptions about vaccines that are supposed to be made available within 6 months, (2) lack of clear border measures to block out citizens and permanent residents so that they would remain outside New Zealand when a deadly disease occurs from a bioweapon scenario and (3) that the IPP was designed only for the flu. Whilst criticisms to improve the IPP may be valid, as authors, we are of the view that these cannot discount the fundamental pillars and building blocks in the IPP that were applied together with other measures to have New Zealand as one of the marvelled global leaders in dealing with the COVID-19 pandemic. Hence, COVID-19 remains another pandemic that will assist in the refinement of the IPP and other related national policies in the future. One cannot throw away the baby with the bathwater. This is more so given that many other countries with similar pandemic plans, such as the United States of America, are faced with the same dilemma (Strang 2020).

Also to the existing pandemic preparedness measures, New Zealand acted swiftly to harness the spread of COVID-19. The Prime Minister started by issuing COVID-19 precautionary measures on 14 March 2020 that led to border closures. This meant the government took the pathway to ‘go hard and go early in the responses to COVID-19 for public health reasons’ (Ministry of Health 2020b:online). The Prime Minister issued a 3-month-long COVID-19 notice under the *Epidemic Preparedness Act* 2006 and proclaimed the COVID-19 Elimination Strategy on 23 March 2020. This proclamation came in to reinforce a package of measures that were instituted from 14 March 2020 that were geared towards a precautionary approach to manage the pandemic. Travellers with visas that were about to expire between 01 April and 09 July 2020 had these automatically extended to late September 2020 (New Zealand Government 2020b).

The COVID-19 Elimination Strategy was developed a month after New Zealand reported its Patient Zero (Ministry of Health 2020c). The strategy focused on eradicating transmission chains and preventing new transmission from imported cases. Non-pharmaceutical strategies outlined included physical distancing, cough and sneezing etiquette, hand hygiene, infection control in the health system, case isolation and quarantine, working from home (WFH), school closures, restricting mass gatherings and border closures. The Prime Minister proclaimed a hard national lockdown when the country still had 102 cases and no deaths, and this quick response earned the country global praise (Cousins 2020). The elimination strategy was favoured as this addressed both the health and the economic side of things. It was envisaged that a quick return to normalcy would minimise damage to the economy. The key message from the government was to eliminate stigmatisation and rally everybody to fight the pandemic. This confirms earlier discussions on language and how the COVID-19 messaging ought to be. It seems New Zealand, a country of about 5 million inhabitants, just got this one right. For example, the message promoting the signing up for the COVID-19 contact tracing app released on 20 May 2020 read ‘Protect yourself, your whānau, and your community. Download the NZ COVID Tracer app’ (New Zealand Government 2020c:online). The questions on how to use the app, privacy and security, accessibility and many more were answered on the official Ministry of Health website (https://www.health.govt.nz/our-work/diseases-and-conditions/covid-19-novel-coronavirus/covid-19-resources-and-tools/nz-covid-tracer-app/questions-and-answers-nz-covid-tracer). The Director General of Health, Dr Ashley Bloomfield’s message further reinforced the messaging, indicating that:

By signing up to this app, you’re helping keep yourself and your family safer and supporting New Zealand to stop the spread of COVID-19. This will ensure we can all return to doing the things we enjoy as soon as possible. (New Zealand Government 2020c:online)

On 16 April 2020, the New Zealand government announced its Initial COVID-19 Māori Response Action Plan (Ministry of Health 2020c) that incorporated the COVID-19 Alert Levels to facilitate the controlled and stepwise opening of the lockdown and economy. The Initial COVID-19 Māori Response Action Plan focused on Phase 3 (Cluster Control) and Phase 4 (Pandemic Management) as outlined in the IPP. The response plan positioned equity at the centre, an aspect that brought into the fore matters of fairness and respect. From the four COVID-19 national alert levels, the country had on the top, Level 4 (lockdown signifying the likelihood of the disease not contained), followed by Level 3 (restrict – High risk as the disease will not have been contained), then Level 2 (reduce – disease contained but with the risk of community transmissions remaining) and, lastly, Level 1 (prepare – the disease is contained).

Under Level 4 (Ministry of Health 2020c), people had to stay at home; travel was severely restricted, businesses and education facilities were closed, healthcare services were reprioritised, with all gatherings cancelled and public venues closed. At Level 3, measures included physical distancing of 2 m outside the homes, the opening of schools for learners between 1 and 10 years of age and people had to work from home, unless where it was not possible. Gatherings of up to 10 people were allowed for funerals, weddings and *tangihanga*. At-risk population, including the elderly and those with medical conditions, had to stay at home as appropriate. During Level 2, there was a further relaxation of lockdown regulations, including permitting gatherings of up to 100 people, sports under strict conditions, opening of some public venues and permitting schooling and tertiary education. Under Level 1 (the time this correspondence was finalised), border entry measures were in place to minimise the risk of importing COVID-19 cases, all basic non-pharmaceutical strategies such as health and hygiene awareness, physical distancing, testing, contact tracing, isolation and quarantine remained. These became the new normal. Restrictions on gatherings were completely lifted, with crowds allowed for sporting events. Restrictions on domestic transportation systems were lifted, and those that had flu-like symptoms were encouraged to stay home.

Following the successful implementation of the highlighted non-pharmaceutical strategies, New Zealand declared COVID-19 elimination and lifted all domestic restrictions on 08 June 2020. This was after no new cases of COVID-19 had been reported for 17 consecutive days and the recovery of the last known patient. This meant moving to the last restricting lockdown level (Level 1) after the country had instituted a 7 weeks hard lockdown. During the May 2020 budget, the Minister of Finance (Robertson 2020) presented a historical health budget indicating upfront that investment in the health sector had become more critical. It emerged that a total of NZ$500 m was allocated before the budget to deal with COVID-19. A further NZ$37m was allocated during the budget speech to sustain laboratory testing capacity, for ambulances, aged care and related services.

## Lessons for other countries

The New Zealand story presents the world with an opportunity to gain insights on early and swift interventions and coordinated government efforts to eliminate COVID-19. It also presents lessons on disaster preparedness as the 2017 IPP provided an existing framework to move fast. The development of the COVID-19 Elimination Strategy, as well as the Initial COVID-19 Māori Response Action Plan with the Alert Levels, was critical. The messaging that eliminated anxiety and stigma amongst the residents was also important (Cousins 2020). In addition, Wilson (2020) sees good pandemic leadership from Prime Minister Jacinda Ardern, who managed to build a shared sense of purpose to minimise COVID-19 damage to lives and livelihoods. The government was willing to listen and be led by experts leading to the ‘flattening of the curve’. Lastly, the gradual and stepwise lockdown easing as per the Alert Levels meant that the pandemic got managed as appropriate.

Nevertheless, the movement of the country to the last Alert Level does not mean that COVID-19 cases may not resurface. This is an aspect the New Zealand government is well aware of. To this end, measures including case detection, isolation, contact tracing and quarantine were left in place. As of 02 September 2020, about 2.1 million New Zealanders had downloaded the COVID Tracer app (Blake-Persen 2020). This figure was about half of those over 18 years and compared with 37% of downloads of similar apps in Ireland and 24% in Australia.

Following the flattening of the curve, there was a mini second wave that witnessed the country recording more active cases in August 2020 following a community outbreak in Auckland (The Guardian 2020). The Auckland cluster grew to 178 cases at some point, resulting in national increases in COVID-19 cases. This resulted in the country getting back into stricter lockdown (Massola 2020). Auckland went back to Level 3, whilst the rest of the country was placed under Level 3 on 12 August 2020. The second set of easing of lockdown restrictions was announced on 21 September 2020 for the rest of the country, apart from Auckland that had to see through another 2 weeks (The Guardian 2020). Once more, the quick return to stricter lockdown as appropriate shows good leadership. The results did not disappoint either, as the country managed to contain the second wave.
